# The Juggernaut of Adaptive Metabolism in Cancers: Implications and Therapeutic Targets

**DOI:** 10.3390/cancers14215202

**Published:** 2022-10-23

**Authors:** Samson Mathews Samuel, Peter Kubatka, Mehdi Shakibaei, Dietrich Büsselberg

**Affiliations:** 1 Department of Physiology and Biophysics, Weill Cornell Medicine-Qatar, Education City, Qatar Foundation, Doha 24144, Qatar; 2 Department of Medical Biology, Jessenius Faculty of Medicine, Comenius University in Bratislava, 03601 Martin, Slovakia; 3 Musculoskeletal & Tumor Biology Research Group, Faculty of Medicine, Institute of Anatomy, Ludwig-Maximilians-University Munich, 80336 Munich, Germany

The disease of cancer instills a sense of fear and dread among patients and the next of kin who are indirectly affected by the deteriorating quality of life of their loved ones [1,2]. Furthermore, the COVID-19 pandemic has significantly increased the prevalence of fear of disease progression, anxiety, and depression among cancer patients [3]. Advancements in modern medicine and booming knowledge regarding the science/molecular mechanisms behind the development and progression of many cancers have led to several significant developments in oncology and its therapeutics. The major achievements include but are not limited to: (1) early cancer diagnosis, (2) efficacious therapeutic intervention, and (3) the ability to predict the outcome of the disease to some extent. However, the rate of incidence of cancers is increasing. Globally, cancers remain a major cause of death, and their impacts on the economic and clinical burden is rising. The urgent need for strategies to prevent cancer development for those at risk and improve treatment strategies for the benefit of those already affected calls for the development of more efficient drugs, better methods of drug delivery, and advancement in patient care to improve the overall quality of life of the affected individuals [4].

While cancers have a hereditary or genetic pre-disposition, they often develop over the years/decades. They are triggered by different processes involving DNA damage, metabolic alterations, epigenetic modifications, chronic inflammation, interactions between aberrant molecular pathways, the inhibition of apoptosis, and cellular crosstalk with the neighboring tissues [4]. Since the 1920s, when Otto Warburg first reported higher rates of aerobic glycolysis, lactate accumulation, and reduced oxidative phosphorylation (Warburg effect) in cancers, the concept that rapidly proliferating cancer cells adapt metabolically to meet their higher energy demands has gained momentum [5,6] (Figure 1). Glucose/carbohydrate metabolism is the most well-studied and targeted adaptation in terms of cancer metabolic alterations [7] (Figure 1). Furthermore, scientific evidence points to the pro-oncogenic and tumor-supportive role of several glycolytic enzymes, proteins, and intermediate substrates (precursors for nucleotide, amino acid, and lipid biosynthesis) of glucose-metabolizing pathways [7,8] (Figure 1). Targeting one or more of these entities by cutting off the glucose-molecule-derived energy and precursor molecule supply should then suppress the cancer cell proliferation, induce cell death, and thus inhibit tumor growth. However, in such a scenario, interestingly, the neoplastic cellular machinery instinctively switches to alternate metabolic pathways (such as glutamine metabolism) to derive cellular energy and maintain the supply of precursors for macromolecular biosynthesis [7,8,9] (Figure 1). More recently, in the neoplastic tissues, other dimensions of the rewired metabolism, such as (1) adaptive changes in amino acid (glutamine), protein, lipid, and nucleotide metabolism; (2) altered propionate metabolism; and (3) isoforms of metabolic-pathway-related enzymes that support tumorigenesis, cancer progression and aggressiveness, and drug resistance, have garnered attention [7,9,10].

In this Special Issue, entitled “Significance of Altered (Glucose) Metabolism in Cancers” (available online: https://www.mdpi.com/journal/cancers/special_issues/metabolism_cancers (accessed on 06 October 2022), we focus on the unique metabolic alterations and adaptations that support cancer cell proliferation and growth, helping these cells to evade programmed cell death. Additionally, we examine how these aberrant metabolic pathways can be targeted to improve cancer treatment.

1. The Content of This Special Issue

This Special Issue of *Cancers* provides a broad and up-to-date overview of the significant aspects that comprise the research on, and developments in, the study and targeting of cancer metabolism for the treatment of different cancers.

Within the framework of this Special Issue, six manuscripts, including one original research article, have been published. One of the review articles was chosen as the editor’s choice article.

In their original article, Gondáš et al. examined the unique ability of cultured human brain cancer cells (human glioma, glioblastoma, and neuroblastoma) to metabolize leucine (a ketogenic amino acid), an essential amino acid required for key cellular metabolic processes [11]. The authors reported that the enzyme 3-methylcrotonyl-CoA carboxylase (MCC), detected in the cultured cancer cells and human tumor samples, gave neoplastic tissue the ability to catabolize leucine and, thus, contribute to nitrogen metabolism in cancer cells and consequently utilize the derived carbon atoms in order to drive the Krebs/tri-carboxylic acid [11]. In fact, the 3-hydroxybutyrate and citrate produced by the cancer cells were efficiently exchanged for metabolites from the microenvironment, which were then utilized for their metabolic requirements, generating lipids and supporting energy metabolism, or they could be utilized as a building material within the cells’ anabolic processes, thus sustaining selective growth and proliferation [11]. More studies are warranted to ascertain whether the leucine metabolism mechanism in neoplastic tissue can be targeted to treat such cancers effectively [11].

In their editor’s choice article, Brockmueller et al. reviewed the ways in which various cancer cells depend on their adaptive/altered glucose metabolism for survival and proliferation [12]. Areas of particular significance were briefly discussed, such as glycolysis and the Warburg effect, and how glycolytic intermediates can be channeled for the synthesis of nucleotides and other macromolecules [12]. A major part of the review is dedicated to the ways in which resveratrol, the red wine polyphenol, can be used as an effective anti-cancer agent targeting multiple pro-oncogenic and anti-apoptotic cellular signaling pathways related to glucose metabolism in several different cancers (breast, lung, colorectal, prostate) [12]. The role of sirtuin proteins as major intracellular targets/mediators of the anti-cancer effect of resveratrol was also discussed in extensive detail [12].

AlZaim et al. focused on the relationship between metabolic disorders and the incidence, risk, increased aggressiveness, and enhanced progression and metastasis of prostate cancer [13]. The review focused on the unique molecular mechanisms involved in the thrombo-inflammatory processes implicated in adipose tissue dysfunction and its relation to prostate cancer [13]. An in-depth review of the role of the periprostatic adipose tissue (PPAT) and pleotropic implications of several coagulation cascade factors, including the tissue factor, thrombin, and active factor X, and their role in driving the development and progression of prostate cancer was provided [13]. The authors also proposed several novel PPAT-related therapeutic targets and possible therapeutic interventions that could effectively reverse the adverse effects of the thrombo-inflammatory processes on prostate cells and reduce the risk and progression of prostate cancer [13]. However, in-depth analyses are required to specify the precise pharmacology and cell signaling pathways of perspective drugs such as direct oral anticoagulants or protease-activated receptor ligands.

The role of cancer stem cells (CSCs) in the development of chemo-resistant breast cancers, thereby supporting the progression, invasion, metastasis, and relapse of breast cancer, was reviewed by Samuel et al. [14]. While delving briefly into the various mechanisms through which CSCs confer chemo-resistance in breast cancers, the article focused on combating CSC-derived drug resistance using the most widely prescribed oral anti-diabetic drug, metformin [14]. In addition to its indirect and direct anti-neoplastic effects, metformin could counteract CSC-conferred multi-drug resistance in breast cancers via AMPK-dependent and independent mechanisms and, thus, re-sensitize breast cancers, making them susceptible to chemotherapeutic interventions [14].

Dysregulated glutamine metabolism, as a hallmark of several cancers, was the focus of the review by Halama A and Suhre K [9]. It is evident that cancer cells derive their energy and maintain their supply of macromolecules for growth, survival, and proliferation by metabolizing glutamine when glucose is restricted in the cancer environment [9]. Hence, targeting and inhibiting glutamine metabolism could play key roles in limiting the growth of a malignant tumor [9]. However, the inhibition of glutamine metabolism in cancers leads to the utilization of alternate metabolic pathways for survival and growth [9]. The authors provided an in-depth review of the role of dysregulated glutamine metabolism in several different cancers, different anti-neoplastic therapeutic targets in glutamine metabolism, alternative metabolic pathways activated upon the inhibition of glutamine metabolism, and the efficacy of combinatory/dual-pathway-targeting therapeutic interventions to improve the efficacy of anti-cancer treatments [9]. In this regard, methods supporting the continuous monitoring of metabolic alterations in the cancer cells could substantially improve cancer management, offering a more personalized medicinal approach to treating cancer patients [9].

Samec et al. in their review, discussed the role of cancer tissue hypoxia in the modulation of several cancer-related genes and metabolic pathways that support cancer cell proliferation, growth, and survival [15]. The review focused on the specific role of hypoxia-inducible factor 1 (HIF-1), a hypoxia-induced transcription factor, and its crosstalk with various enzymes and regulatory aspects of glucose metabolism in cancers, discussing the efficacy of several HIF-1 inhibitors in inhibiting cancer cell proliferation and survival [15]. Naturally occurring compounds, such as flavonoids, have gained importance as effective anti-inflammatory and anti-cancer agents. Recent oncology research has described numerous flavonoids and their derivatives as potent inhibitors of HIF-1, being associated with the modulation of important components of glycolysis in cancer cells, i.e., pyruvate kinase M2 (PKM2), glucose transporters (GLUTs), lactate dehydrogenase (LDHA), phosphofructokinase-1 (PFK-1), hexokinase II (HKII), or pyruvate dehydrogenase kinase (PDK). Finally, the authors reviewed the oncostatic potential of different flavonoids in cancer cells by regulating the activity and function of HIF-1, consequently shedding light on how this can influence its role in the Warburg effect observed in other cancer types [15].

2. Targeting Metabolic Plasticity in Growing Tumors

The study of metabolic adaptations, as hallmarks of a growing malignant tumor and their links to cancer progression, aggressiveness, drug resistance, invasion, and metastasis, is a rapidly advancing field in cancer research. Unlike previous notions, it is evident that, apart from altered glucose metabolism (the Warburg effect) and high rates of glycolysis, the metabolic plasticity of cancer cells enables them to adapt to a wide range of challenging and metabolically adverse tumor microenvironments, which may otherwise be considered be detrimental to a normal cell. Many different aspects of adaptive tumor metabolism, such as altered metabolic enzyme expression and function, increasing dependence on/the differential utilization of certain amino acids (such as glutamine, serine, and branched-chain amino acids), altered de novo lipid synthesis, aberrant nucleotide synthesis, and the influence of the metabolism of tumor-associated normal cells, have come to light owing to the scientific work of cancer scientists across the globe.

Interestingly, the metabolic symbiosis between normal and cancerous cells has been shown to influence cancer cell proliferation and tumor growth. For instance, the lactate produced/released by cancer-associated fibroblasts can be utilized to drive the TCA cycle in the cancer cells in the vicinity [16]. Furthermore, the lactate produced/released by the hypoxic regions of a growing tumor activate cellular respiration in well-oxygenated tumor cells [16]. In such a scenario, since lactate utilization by the tumors is dependent on the expression of monocarboxylate transporters (MCT), targeting MCTs (which are highly expressed in a variety of tumors) should be an exciting strategy to curb tumor growth [16]. An improved understanding of the metabolic interactions between tumor cells and tumor-associated normal cells, therefore, should play a critical role in drug development, targeting, and delivery for the treatment of cancers. De novo drug development and testing are, however, time-consuming processes. Hence, the re-purposing of drugs and use of naturally occurring (phytochemical) compounds with proven abilities to target various aspects of tumor metabolism are encouraged.

Metabolic dependency varies widely between different types of cancers/tumors and, hence, from patient to patient. It can be significantly influenced by the tumor microenvironment, cancer lineage, and the various epigenetic and genetic events that occur in cancer cells [16]. Hence, the challenge lies in the fact that a particular drug targeting a specific altered metabolic activity or enzyme of cancer cells may work effectively in curbing tumor growth in one patient. In contrast, another patient may be less responsive to the same therapeutic intervention [16]. Tumors that rely on aerobic glycolysis (glucose-metabolism-dependent) for proliferation and survival should thus be susceptible to agents that target glucose uptake/utilization and inhibit tumor angiogenesis [17]. On the other hand, while agents targeting glutamine metabolism should be effective in curbing tumor growth in cancers dependent on the Krebs cycle (TCA cycle), the impaired mitochondrial electron transport chain and oxidative phosphorylation can be targeted by drugs that interfere with reductive carboxylation and fatty acid synthesis [17].

The drawback of targeting a single altered metabolic process in tumors is that the cancer cells adapt in order to survive by switching their metabolic dependence to other alternate signaling pathways in such a scenario. Consequently, combined treatments with natural, non-toxic, multi-target drugs that act on multiple cellular survival signaling pathways may be more efficient than therapy based on mono-target chemotherapeutic agents in treating cancer. Furthermore, the efficacy of anti-metabolite targeting may rely on the time and duration of drug administration (when tumor cells have a greater dependence on specific enzymes than normal tissues), dosage, number of administered doses, and spacing of the drug administration (when multiple doses are necessary) [17]. It is, therefore, essential to identify patients who are most likely to respond to a given therapeutic intervention on a case-by-case basis (predictive, preventive, and personalized medicine, or 3PM).

## Figures and Tables

**Figure 1:**
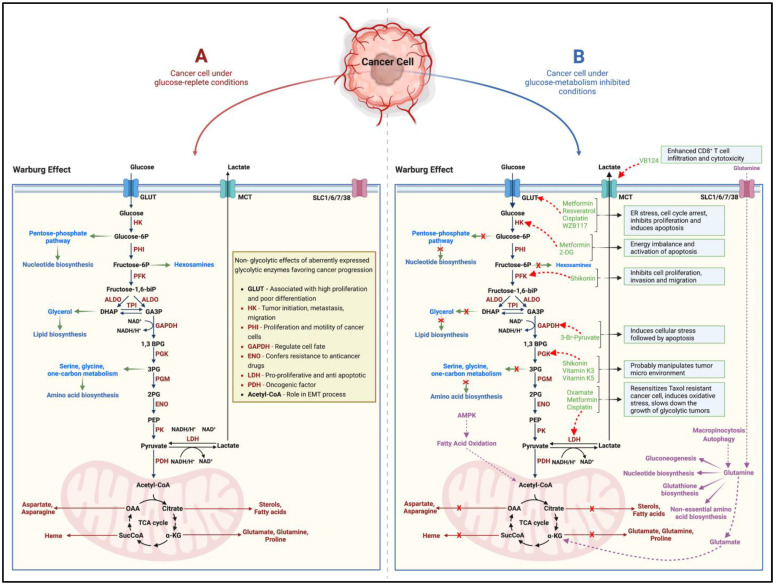
Schematic (**A**) indicates the altered glucose metabolism (Warburg effect) under the effect of glucose in cancer cells. While several of the glycolytic intermediates replenish precursor molecules for macromolecular biosynthesis in the rapidly growing tumor, alterations in the expression and function of metabolic enzymes have been shown favor cancer progression in several ways. Several pharmacological agents, naturally occurring compounds, and small-molecule inhibitors (**B**) effectively block the tumorigenic ability of the target enzymes and metabolic pathways. However, the cancer cell can adapt to utilize glutamine metabolism as an alternate pathway to derive cellular energy and maintain the supply of precursors for macromolecular biosynthesis. The black and brown arrows indicate the pathway and flow of components involved in the pathway. The green arrows indicate how glycolytic intermediates can act as precursor molecules for the biosynthesis of various macromolecules. The red ‘x’ marks indicate how inhibiting glucose metabolism in cancer cells shuts off the supply of precursor molecules for macromolecular biosynthesis. The red dotted arrows indicate the inhibitory nature of the pharmacological agents, naturally occurring compounds, and small-molecule inhibitors on the target enzymes/proteins. The purple arrows alternate/adaptive metabolic pathways that support growth and proliferation of cancer cells. HK = Hexokinase, PHI = Phospho-hexose isomerase, PFK = Phosphofructokinase, ALDO = Aldolase, TPI = Triose-phosphate isomerase, GAPDH = Glyceraldehyde phosphate dehydrogenase, PGK = Phosphoglycerate kinase, PGM = Phosphoglycerate mutase, ENO = Enolase, PK = Pyruvate kinase, LDH = Lactate dehydrogenase, PDH = Pyruvate dehydrogenase, GLUT = Glucose transporter, MCT = Monocarboxylate transporter, SLC = Solute carrier transporter. Created with BioRender.com.
